# Plant microbiome technology for sustainable agriculture

**DOI:** 10.3389/fmicb.2024.1500260

**Published:** 2024-11-13

**Authors:** Muhammad Sajid Hanif, Muhammad Tayyab, Elamin Hafiz Baillo, M. Mominul Islam, Waqar Islam, Xiaofang Li

**Affiliations:** ^1^Centre for Agricultural Resources Research, Institute of Genetics and Developmental Biology, Chinese Academy of Sciences, Shijiazhuang, China; ^2^University of Chinese Academy of Sciences, Beijing, China; ^3^Institute of Marine Sciences and Guangdong Provincial Key Laboratory of Marine Biotechnology, Shantou University, Shantou, China; ^4^Agricultural Research Corporation (ARC), Ministry of Agriculture, Wad Madani, Sudan; ^5^Xinjiang Key Laboratory of Desert Plant Roots Ecology and Vegetation Restoration, Xinjiang Institute of Ecology and Geography, Chinese Academy of Sciences, Urumqi, China

**Keywords:** plant microbiome, microbial diversity, growth promotion, microbiome-based farming approaches, sustainable agriculture

## Abstract

Plants establish specific interactions with microorganisms, which are vital for promoting growth and resilience. Although advancements in microbiome modulation technologies show great potential for sustainable agriculture, several challenges have hindered the wider application of plant microbiomes in the field. These challenges may include inconsistent microbial colonization, competition with native microbiota, and environmental variability. Current strategies, while promising, often yield inconsistent results in real-world agricultural settings, highlighting the need for more refined approaches. Agricultural practices and plant genotypes significantly influence the composition and function of plant-associated microbiota. A data-driven strategy that incorporates genomic profiling, environmental assessments, and optimized delivery systems is essential for selecting effective microbial strains. Additionally, refining farming practices, such as crop rotation, intercropping, and reduced tillage, along with robust plant breeding programs, can greatly enhance crop health and productivity.

## 1 Introduction

Over the past few decades, research has unveiled the intricate and essential role of the plant microbiome in supporting plant growth, health, and resilience. The plant microbiome, encompassing diverse microbial communities associated with plant organs, includes bacteria, fungi, and archaea, with bacterial components being the most studied (Buée et al., 2009; Vorholt, 2012; Reinhold-Hurek et al., 2015). These microbial communities interact with the plant as a holobiont, which plays a critical role in maintaining plant productivity, particularly under changing environmental conditions (Berg et al., 2016; Brader et al., 2017; Lemanceau et al., 2017; Hassani et al., 2018).

As agriculture faces growing challenges such as climate change, resource depletion, and the increasing demand for sustainable practices, traditional reliance on chemical inputs like pesticides and fertilizers has raised concerns about their long-term environmental and economic impacts (Bhattacharyya and Jha, 2012). In response, the plant microbiome has emerged as a promising alternative to these inputs, offering a biologically driven approach to enhancing crop health and productivity. Microorganisms, particularly plant growth-promoting bacteria (PGPB), have demonstrated the capacity to improve nutrient uptake, stimulate plant growth, and enhance resistance to pathogens, positioning them as valuable tools for sustainable agriculture (Mitter et al., 2016). This review provides an up-to-date discussion on the potential of plant microbiome technology in addressing current challenges facing modern agriculture, with a focus on bacteria. To this end, we first summarize the knowledge on the diversity and functionality of the plant microbiome, and finally propose approaches to harness the microbiome for sustainable agriculture.

## 2 Plant microbiome: functionality and diversity

### 2.1 Belowground plant microbiome

Plants recruit their microbiome from various environmental reservoirs, including the rhizosphere, phyllosphere, spermosphere, anthosphere, and carposphere (Hardoim et al., 2015). The root microbiome is mainly horizontally transferred; that is, it comes from the soil environment, containing different microorganisms (Shade et al., 2017). Nevertheless, bacteria can also transmit vertically through seeds (Fierer, 2017). Microorganisms thrive in developing plant roots, and seeds are an important source of them (Truyens et al., 2015; Nelson, 2018). Plant root system presents a unique environmental niche for the soil microbial community, which settles in the root, rhizosphere and aerial part (Berendsen et al., 2012). One of the most significant mutualistic relationships in the root microbiome is the nodule formation between leguminous plants and nitrogen-fixing bacteria such as *Rhizobium* (Liu et al., 2021; Granada Agudelo et al., 2023). These nodules enhance nitrogen availability, which is fundamental to plant functionality and growth (Lindström and Mousavi, 2020), ultimately improving soil fertility and supporting sustainable agriculture (Ojija and Aloo, 2023). Nodule formation represents a classic example of a mutualistic interaction where both the plant and the microbes benefit (Haldar and Sengupta, 2015).

The rhizosphere, a thin layer of soil directly influenced by plant roots, is regarded as a hot zone of microbial activity and one of the most complicated ecosystems (Raaijmakers et al., 2009). Donn et al. (2015) demonstrated that bacterial composition of wheat rhizosphere changed across years and noticed the abundance of *Pseudomonads* and *Actinobacteria* was 10-fold greater in rhizosphere than that of bulk soil. Root exudates (e.g., amino acids, fatty acids, organic acids, phenolics, nucleotides, plant growth regulators, sugars, putrescine, vitamins, and sterols) are leading factors in terms of shifting microbial composition around roots, the so-called rhizosphere effect (Mendes et al., 2013; Rasmann and Turlings, 2016; Sasse et al., 2018; Zhalnina et al., 2018; Jiang et al., 2019). For example, benzoxazinoids (BXs), a class of defensive secondary metabolites attributed to maize roots, had a critical role in changing root-associated microbiota composition. Actinobacteria and Proteobacteria are shown to be affected by BXs compounds (Hu et al., 2018). Overall, phenotype and genotype variations in plant traits are the definitive explanation for this hypothesis, which can guide specific microbial communities.

**Figure 1:**
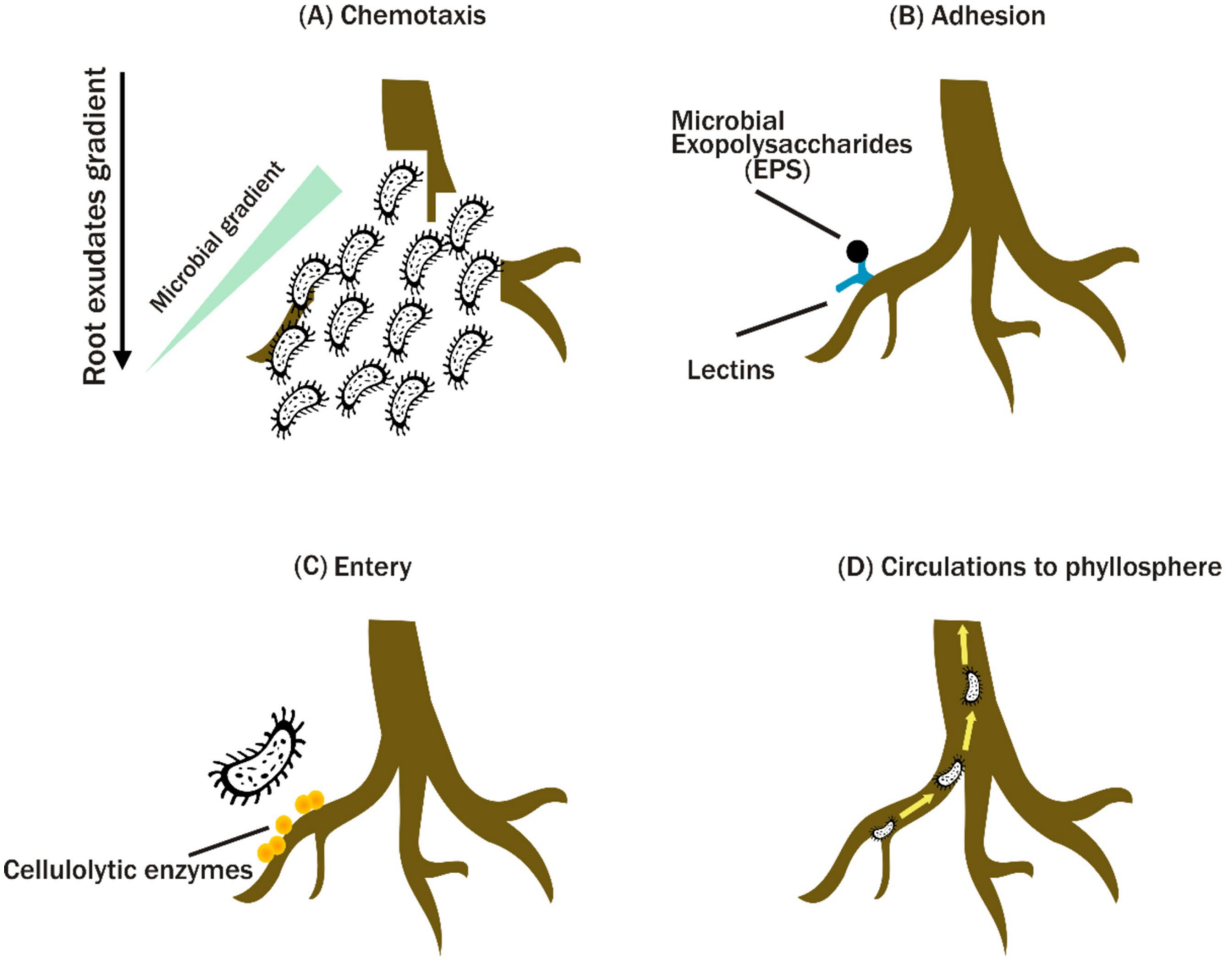
Mechanisms of plant microbiome colonization. Microbial recruitment in the rhizosphere is initiated by chemotaxis toward plant exudates (A). Microbes adhere to plant lectins via exopolysaccharides (EPS) (B), penetrate root tissues with the aid of cellulolytic enzymes (C), and disseminate throughout the plant system (D).

A vast range of bacterial endophytes can successfully invade plant roots. Bacterial endophytes frequently enter root tissues via passive processes such as root fractures or lateral root emergence points, as well as active mechanisms (Kandel et al., 2017) (Figure 1). Endophytes’ colonization and transmission within plants are impacted by various factors, such as plant resource allocation and the endophytes’ colonization ability. Various bacterial taxa have been found to be able to colonize inside root tissues, such as Acidobacteria, Proteobacteria, Verrucomicrobia, Bacteroidetes, Chloroflexi, Planctomycetes, Gemmatimonadetes and Firmicutes which commonly occur in grapevine roots (Burns et al., 2015; Samad et al., 2017a; Correa-Galeote et al., 2018). Additionally, the study by Correa-Galeote et al. (2018) found that Proteobacteria, Firmicutes and Bacteroidetes are the predominant phyla within the maize roots and the history of soil cultivation substantially influenced the abundance of these phyla.

### 2.2 Microbiota in above-ground plant

Above-ground plant tissues (e.g., vegetative foliar portions, floral parts, and leaves) provide distinct habitats for epiphytes and endophytes; yet, the ecology of endosphere and episphere bacteria differs significantly. Most endophytes propagate systemically via the xylem to various plant compartments, but they can also reach plant tissues through aerial plant parts (Compant et al., 2010, 2011). Different above-ground plant compartments host distinct endophytic communities, influenced by the allocation of plant resources. According to observations, phyllobacteria also originated in the soil environment, affected by environmental and plant factors (Vorholt, 2012; Wallace et al., 2018).

Various microorganisms are found at the species and genus levels in the endosphere and phyllosphere. For example, the structural analysis of grapevine carposphere or phyllosphere microbiome revealed that the predominant genera were *Sphingomonas*, *Frigoribacterium*, *Pseudomonas*, *Bacillus*, *Curtobacterium*, *Acinetobacter*, *Enterobacter*, *Citrobacter*, *Erwinia*, *Methylobacterium* and *Pantoea* (Campisano et al., 2014; Kecskeméti et al., 2016; Wallace et al., 2018). Recently, Wallace et al. (2018) investigated the leaf microbiome of 300 various maize lines. They found that *Methylobacteria* and *Sphingomonads* were the predominant taxa. They also found that environmental variables mainly drove the microbial composition of the phyllosphere. The dominant taxa are found to be *Pseudomonas* and *Enterobacteriaceae* in apple flowers (Steven et al., 2018). Similarly, a number of studies have found *Pseudomonas* to be the most abundant genus in apples, almonds, grapefruit, tobacco and pumpkin flowers (Aleklett et al., 2014). In general, microbiota on and within the plant tissue originate mainly from seed, soil, and air, whose composition of communities was determined by several factors (e.g., environmental, farm management techniques, and soil). The unique assembly in host and compartments indicates that the plant has a strong functional interaction with its above-ground microbiota, but further research is needed to better understand the mechanisms governing this relationship. Above-ground microbiomes and endophytes are well known for their role in improving plant growth, stress mitigation, and disease resistance (Hassani et al., 2018; Stone et al., 2018).

### 2.3 Core and satellite microbiomes

Microorganisms that are consistently associated with specific plant species or genotypes, irrespective of environmental fluctuations, form the core plant microbiome (Toju et al., 2018). These core taxa are considered functionally critical due to their stable presence. Evolutionary selection has equipped them with essential genes that contribute to plant fitness. Notable examples include *Bradyrhizobium*, *Sphingobium* and *Microvirga* in potatoes (Pfeiffer et al., 2017), as well as *Pseudomonadaceae*, *Micrococcaceae* and *Hyphomicrobiaceae* in grapevines (Zarraonaindia et al., 2015). Keystone taxa within the core microbiome are pivotal in maintaining ecosystem stability and promoting plant health, primarily through nutrient cycling, pathogen suppression, and growth enhancement mechanisms (Lemanceau et al., 2017). Satellite taxa play critical roles in functions such as disease suppression and nutrient cycling, contributing disproportionately to the system’s overall stability (Hanski, 1982; Gera Hol et al., 2015). In contrast, satellite taxa are less abundant, displaying a transient presence across fewer environments. Although these taxa may not be as ubiquitous, their ecological contributions are significant, particularly in enhancing ecosystem resilience and stability (Jousset et al., 2017). Examples of satellite species include *Lipomyces* sp. (Kunito et al., 2012) and *Cladorrhinum* sp. (Tayyab et al., 2021), which contribute to aluminum toxicity reduction, plant growth promotion, and biological control.

Despite their transient and often rare nature, rare microorganisms are integral to the flexibility of the microbial community, enabling rapid responses to environmental perturbations. The concept of the “rare biosphere” is especially relevant here, as rare microbial taxa contribute to microbial diversity and the capacity of ecosystems to withstand biotic and abiotic stresses (Lynch and Neufeld, 2015).

**Figure 2:**
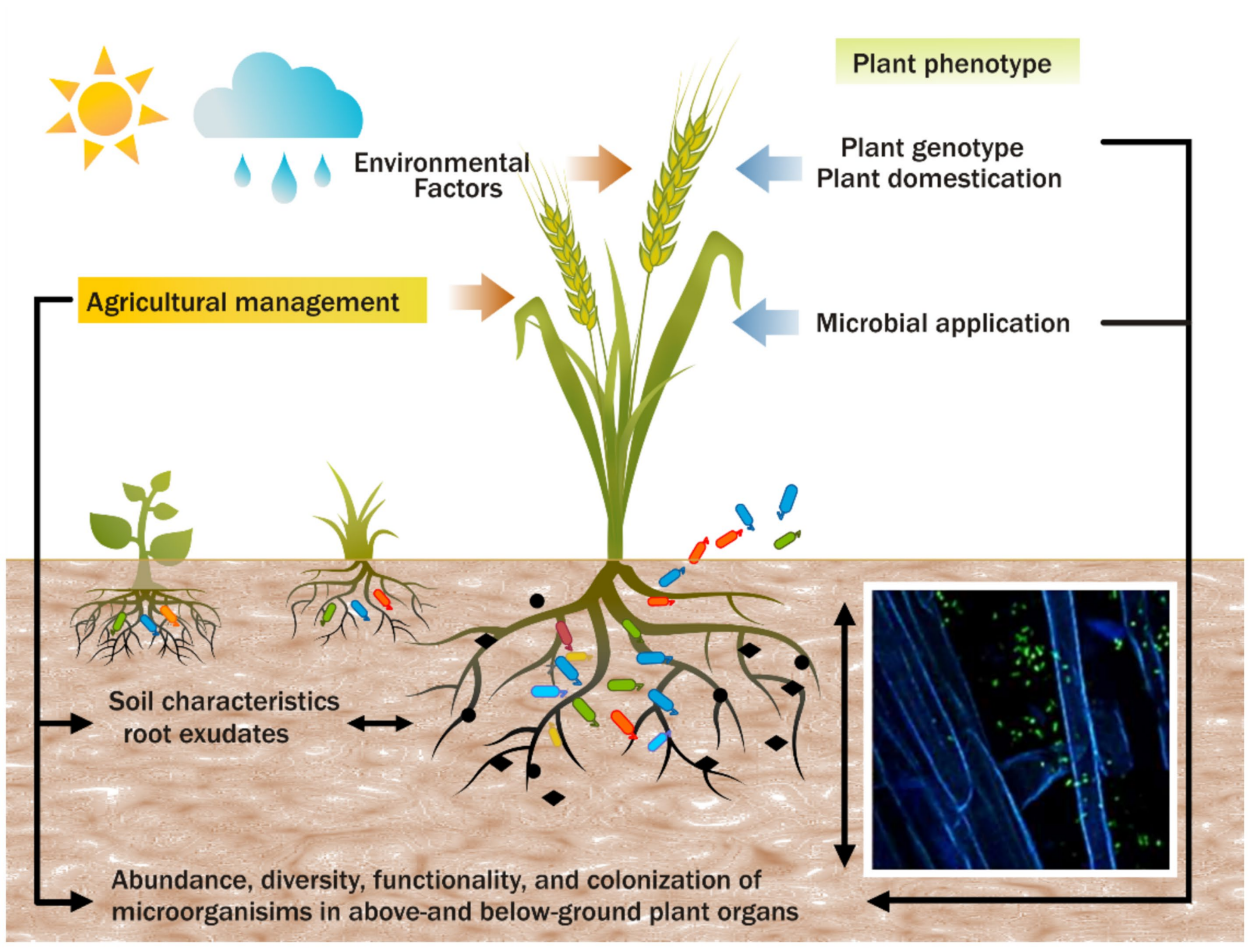
The complex interactions between plant genotypes, microbiota, and agricultural practices that influence plant phenotypes and traits.

The interplay between core and satellite microbiomes is essential in understanding the functional dynamics of plant health. Core taxa offer stability, providing critical support under standard environmental conditions. In contrast, satellite taxa introduce ecological flexibility, enabling the plant microbiome to adapt to environmental shifts. This synergistic interaction is fundamental to plant resilience, particularly in agriculture, where crops are exposed to diverse stresses. Case studies involving microbial inoculants have shown that targeting core taxa for long-term benefits, along with managing satellite taxa for short-term adaptations, can significantly enhance agricultural productivity and disease resistance (Compant et al., 2019). Understanding and leveraging the interactions between core and satellite microbiomes are vital for improving plant fitness and ecosystem health, especially in the face of environmental change and agricultural intensification (Figure 2).

## 3 Functions of plant microbiota

The plant microbiota encompasses a wide variety of microorganisms, including beneficial, neutral, and pathogenic species, each playing distinct roles in plant health and development. Plant growth-promoting bacteria (PGPB) form a crucial subset of the microbiota, promoting plant growth through various mechanisms.

### 3.1 Biological nitrogen fixation

Biological nitrogen fixation (BNF) is a key process wherein plant-associated bacteria convert atmospheric nitrogen into a usable form of ammonia for plants. This symbiotic relationship primarily occurs in leguminous plants through specialized root nodules that harbor nitrogen-fixing bacteria, such as *Rhizobium* and *Bradyrhizobium* (Irisarri et al., 2021; Prüß, 2022). Nitrogen fixation reduces the need for synthetic nitrogen fertilizers which are environmentally detrimental (Mahmud et al., 2020; Soumare et al., 2020). Furthermore, non-nodular bacteria like *Gluconacetobacter diazotrophicus* have expanded the scope of BNF to non-leguminous plants (Dent, 2018). Integrating nitrogen-fixing bacteria into sustainable agriculture reduces greenhouse gas emissions and enhances crop resilience, especially under drought conditions (Minamisawa, 2023).

### 3.2 Mineral solubilization

Microorganisms also facilitate the solubilization of key nutrients like phosphorus and potassium, which are often unavailable in their natural forms. Phosphate-solubilizing bacteria (PSB) convert insoluble phosphate into bioavailable forms through organic acid production (Tahir et al., 2018). Similarly, potassium-solubilizing bacteria (KSB) enhance the availability of potassium, critical for plant water regulation and enzyme activation (Balakrishnan et al., 2021; Mei et al., 2021). For example, *Bacillus megaterium* and *Pseudomonas fluorescens* have been shown to effectively solubilize phosphate, improving plant growth in phosphorus-deficient soils (Patel et al., 2016).

### 3.3 Phytohormone production

Plant-associated bacteria, particularly from the genera of *Bacillus* and *Pseudomonas*, are capable of producing phytohormones such as auxins, cytokinins, and gibberellins. These hormones regulate critical aspects of plant development, including cell division and elongation. *Bacillus megaterium* is a prolific producer of indole-3-acetic acid (IAA), a key auxin that enhances root growth and nutrient uptake (Ahmed and Hasnain, 2010; Tanveer and Ali, 2022). In addition, cytokinins produced by *Pseudomonas* species promote shoot development and delay leaf senescence, contributing to plant health under stress conditions (Shahraki et al., 2022).

### 3.4 Phytohormone regulation through ACC deaminase activity

Ethylene, a plant hormone produced in response to stress, can inhibit plant growth. Certain bacteria mitigate the negative effects of ethylene by producing the enzyme 1-aminocyclopropane-1-carboxylate (ACC) deaminase, which breaks down ACC, the ethylene precursor. For instance, *Pseudomonas stutzeri* A1501 facilitates rice growth under saline conditions by reducing ethylene levels (Han et al., 2015). These ACC deaminase-producing bacteria not only promote plant growth but also improve tolerance to environmental stresses like drought and salinity (Glick and Nascimento, 2021; Ali et al., 2023).

### 3.5 Siderophore activity

Siderophores are iron-chelating compounds produced by bacteria and fungi to solubilize and transport iron in iron-limited environments. *P. fluorescens* and *B. subtilis* produce siderophores such as pyoverdine and bacillibactin, which improve iron acquisition in plants (Nithyapriya et al., 2021; Zhang et al., 2022). This activity not only enhances plant growth but also provides biocontrol against pathogens by limiting their access to iron, an essential nutrient (Lyng et al., 2023; Celina et al., 2024).

### 3.6 Exopolysaccharide and biofilm formation

Exopolysaccharides are high-molecular-weight polysaccharides secreted by bacteria that form biofilms. EPS production facilitates bacterial adherence to plant roots, enhancing nutrient and water uptake. For example, *Paenibacillus polymyxa* produces EPS that aids in wheat root colonization and improves nutrient absorption (Yegorenkova et al., 2013). Biofilms protect the microbial community from environmental stresses, such as desiccation and salinity, and can also protect against pathogens by forming a physical barrier and competing for resources (Bokhari et al., 2021).

## 4 Factors governing microbial community composition

The composition of plant-associated microbial communities is shaped by a dynamic and multifaceted interplay of environmental factors, host-derived elements, and microbial interactions (Turner et al., 2013; Pang et al., 2019; Tayyab et al., 2022). Environmental conditions, including soil type, pH, temperature, moisture, and salinity, significantly influence microbial diversity (Hassani et al., 2018; Tayyab et al., 2021). The microbiome composition varies markedly between plant species, reflecting distinct microbial populations even in closely situated plants of phylogenetically distant species. For instance, grapevines and adjacent weeds exhibit species-specific microbial clades such as *Pedobacter* and *Rhizobiaceae* (Samad et al., 2017b). Experiments in controlled environments further demonstrate that the root microbiome composition in *Ceanothus thyrsiflorus*, *Baccharis pilularis*, and *Pinus muricata* reflects their respective native soils, underscoring the role of plant species identity in shaping microbial communities (Jones et al., 2019). The plant genotype plays a critical role in determining microbial community structure. Differential microbial abundances have been associated with specific plant genotypes, such as in wheat varieties where chromosomal substitutions affect rhizosphere microflora (Neal et al., 1970). Genetic modifications, such as the introduction of specific genes, can substantially alter microbial communities, as demonstrated in studies involving *Sorghum bicolor* and *Arabidopsis thaliana* (Bressan et al., 2009; Chaparro et al., 2014). Notably, certain sugarcane cultivars influence bacterial richness, with cultivars like “Haizhe 22” showing distinct beta diversity patterns and enriching bacterial genera (Tayyab et al., 2022). Plant exudates, such as amino acids, organic acids, sugars, and phenolic compounds, significantly influence microbial colonization and diversity (Sundaram et al., 2015; Olanrewaju et al., 2019; Arafat et al., 2020). Additionally, volatile compounds like methanol produced by plants contribute to the growth of phyllosphere microorganisms (Galbally and Kirstine, 2002; Uhlik et al., 2013). Plant health and immunity mechanisms, such as such as pattern recognition receptors (PRRs) responding to microbe-associated molecular patterns (MAMPs) and damage-associated molecular patterns (DAMPs) help regulate community composition through pathways like pattern-triggered immunity (PTI) and effector-triggered immunity (ETI) (Boller and Felix, 2009; Dodds and Rathjen, 2010; Monaghan and Zipfel, 2012). Temporal shifts in microbial communities across plant developmental stages have been observed, with notable changes in the abundance of groups such as Actinobacteria, Acidobacteria, Bacteroidetes and Cyanobacteria (Chaparro et al., 2014; Chen et al., 2019). These shifts reflect the evolving requirements and interactions between plants and their microbial partners throughout the plant life cycle.

## 5 Harnessing the plant microbiome

### 5.1 Microbial inoculation

Microbial inoculation, central to agricultural microbiology, begins with the meticulous screening of strains for plant growth-promoting (PGP) traits under controlled conditions. Key traits include phosphate solubilization, nitrogen fixation, siderophore production, and plant hormone regulation. Fungal strains such as *Purpureocillium* sp. strain YZ1 have also been shown to modulate plant elemental uptake (Zheng et al., 2021). Strains that perform well in lab assays undergo greenhouse trials and subsequent field evaluations. Although microbial inoculants like *Azospirillum brasilense* Ab-V5 have demonstrated significant yield increases in maize and wheat under optimized conditions (Hungria et al., 2010), field efficacy remains variable, highlighting their potential when conditions are optimized (Fukami et al., 2016; Backer et al., 2018). Factors such as competition with native microflora, suboptimal strain selection, and environmental variables can undermine inoculant performance (Samad et al., 2017a). Formulation, including cell viability and niche compatibility, is crucial for sustained colonization and function (Chowdhury et al., 2015). Biocontrol agents like *B. amyloliquefaciens* FZB42 require early establishment and niche adaptation to trigger effective plant defenses (Chowdhury et al., 2015).

### 5.2 Leveraging microbial consortia

Emerging approaches advocate for using microbial consortia rather than single-strain inoculants to enhance reliability under diverse field conditions (Parnell et al., 2016; De Vrieze et al., 2018) Consortia offers complementary PGP effects and pathogen suppression, enhancing plant health and resilience (Berendsen et al., 2018). For example, *P. consortia* have demonstrated enhanced nutrient assimilation and stress resilience in plants (Guttieri et al., 2015; Hu et al., 2016). Tailoring consortia to specific environmental conditions and plant genotypes further enhances their efficacy, as shown by the host preference of *Azospirillum* (Chamam et al., 2013; Edwards et al., 2015; Santos-Medellín et al., 2017). Nonetheless, ongoing research is necessary to refine consortia selection and optimize their field performance (Herrera Paredes et al., 2018).

### 5.3 Innovations in formulation and delivery

Effective microbial formulations are essential for maintaining inoculant viability and efficacy in the field. Techniques such as encapsulation and surfactant addition improve adherence to plant surfaces, reducing environmental stresses like drift and UV exposure (Preininger et al., 2018; Timmusk et al., 2018). Innovations such as seed microbiome modulation through flower spray inoculation hold promise for improving microbial colonization and plant growth throughout the crop cycle (Pfeiffer et al., 2017). Immobilization technology can be promising to enhance field efficacy of microbial inoculants (Wu et al., 2021).

### 5.4 *In situ* microbiome engineering

The manipulation of existing natural microbial communities *in situ* is an important approach to improve plant health and productivity, which leverages the inherent diversity and functionality of microbial populations already present in the soil and plant environments. One effective strategy within this framework is the selective enhancement of beneficial microbial taxa through practices such as crop rotation, cover cropping, and organic amendments. These practices can promote the proliferation of beneficial microbes while suppressing pathogenic species, thereby enhancing plant resilience to stressors such as drought and disease (Carmen Orozco-Mosqueda et al., 2018; Wang and Haney, 2020).

*In situ* microbiome engineering also includes the application of microbial inoculants that are specifically tailored to enhance the native microbiome. For example, research has shown that inoculating crops with beneficial microbes can lead to significant increases in yield and stress tolerance (Lawson et al., 2019; Yang et al., 2023). Furthermore, the manipulation of root exudates through plant breeding or management practices can selectively recruit beneficial microbes, thereby optimizing the plant’s microbiome for enhanced performance (Clouse and Wagner, 2021; Nadarajah and Natasha Rahman, 2023).

### 5.5 Innovative systems

Design of novel microbial systems is an emerging approach in microbiome engineering. Two notable systems in this category are the Intraspecies Cross Environmental (ICE) system and the Combinatorial CRISPR Array-Guided Engineering (CRAGE) system.

The ICE system focuses on creating synthetic microbial consortia that can thrive across different environmental conditions. By selecting and combining microbial strains that exhibit complementary traits, researchers can develop robust microbial communities capable of enhancing plant growth under varying stress conditions. This approach allows for the fine-tuning of microbial interactions to optimize nutrient cycling and plant health (Uroz et al., 2019; Afridi et al., 2022). For instance, synthetic consortia can be engineered to improve nitrogen fixation or phosphorus solubilization, directly benefiting plant growth (Kaul et al., 2021).

The CRAGE system utilizes CRISPR technology to engineer microbial genomes for specific functions that benefit plant health. By targeting genes involved in beneficial traits such as stress tolerance, nutrient uptake, or pathogen resistance, researchers can create microbial strains with enhanced capabilities. This method not only allows for precise modifications but also facilitates the study of gene function in microbial communities, paving the way for more effective microbiome engineering strategies (Dastogeer et al., 2022; Malayejerdi and Pouresmaeil, 2020).

## 6 Harnessing plant microbiomes through agricultural approaches

### 6.1 Optimizing plant selection for enhanced microbial relationships

Incorporating plant microbiomes into crop breeding programs represents a crucial frontier in enhancing stress tolerance, nutrient uptake, and productivity (Haichar et al., 2008; Bakker et al., 2012; Ofek et al., 2014; Wei and Jousset, 2017) (Figure 2). Different genotypes foster distinct microbial associations, yet the genetic mechanisms driving these interactions remain poorly understood. Domestication has reduced plant genetic and microbial diversity, limiting the ability of modern crops to interact with beneficial microbes (Haudry et al., 2007; Bitocchi et al., 2013; Gopal and Gupta, 2016). However, recent studies reveal significant differences in microbial diversity and composition across sugarcane cultivars, highlighting the potential to select cultivars that promote beneficial microbial associations (Tayyab et al., 2022). Integrating microbiome considerations into breeding practices is pivotal for advancing sustainable agriculture and optimizing productivity (Li et al., 2024).

### 6.2 Effects of agricultural practices on plant-microbe dynamics

Agricultural practices, such as intercropping, organic farming, and reduced tillage, significantly influence plant-microbe interactions (Sánchez-Cañizares et al., 2017) (Figure 2). Practices like organic farming can enhance microbial diversity and abundance, improving ecosystem resilience and plant health (Hartmann et al., 2015; Hartman et al., 2018). For example, sugarcane–legume intercropping has been shown to enhance soil fertility and microbial diversity without compromising crop yields (Pang et al., 2022). The use of bacterial biological control agents also shows promise, with studies demonstrating improved soil chemistry, enriched beneficial microbial communities, and enhanced crop productivity in ratoon sugarcane (Fallah et al., 2023).

## 7 Conclusions and perspectives

Plant microbiomes play a crucial role in sustainable agriculture by fostering diverse and beneficial microbial communities that contribute to plant health and productivity. While the significance of plant-associated microbiomes has been recognized for centuries, it was not until the 1980s that substantial progress was made in understanding and harnessing their potential. Although microbial inoculants have been developed to enhance plant growth and resilience, their effectiveness in real-world agricultural settings has been inconsistent (Kimotho et al., 2024). This underscores the urgent need for targeted strategies to optimize microbial selection, formulation, and delivery methods to improve field performance.

Tailoring microbial consortia to specific crops and environmental conditions shows great promise in overcoming current challenges. Techniques like organic farming and intercropping can be employed to promote beneficial microbial interactions and enhance plant health (Wang and Li, 2019). Moreover, advancements in next-generation plant breeding that focus on facilitating favorable plant-microbe relationships offer exciting possibilities for boosting crop productivity and sustainability.

Future research should focus on filling knowledge gaps related to how inoculants impact existing microbiomes, the functional dynamics within complex microbial communities, and the factors influencing microbial colonization in agricultural settings. A comprehensive understanding of these processes is essential for developing resilient microbial inoculants and consortia that can thrive in diverse farming environments. The development of tailored formulations and delivery systems suited to the specific requirements of crops and local conditions is vital for achieving success in the field. Innovative approaches such as encapsulation technologies and seed microbiome modulation show promise in enhancing microbial colonization and persistence, bridging the gap between laboratory research and practical application in agricultural settings.

## Data Availability

The original contributions presented in the study are included in the article/supplementary material, further inquiries can be directed to the corresponding author.
